# Assessment of mortality and hospital admissions associated with confirmed infection with SARS-CoV-2 Alpha variant: a matched cohort and time-to-event analysis, England, October to December 2020

**DOI:** 10.2807/1560-7917.ES.2022.27.20.2100377

**Published:** 2022-05-19

**Authors:** Gavin Dabrera, Hester Allen, Asad Zaidi, Joe Flannagan, Katherine Twohig, Simon Thelwall, Elizabeth Marchant, Nurin Abdul Aziz, Theresa Lamagni, Richard Myers, André Charlett, Fernando Capelastegui, Dimple Chudasama, Tom Clare, Flavien Coukan, Mary Sinnathamby, Neil Ferguson, Susan Hopkins, Meera Chand, Russell Hope, Meaghan Kall, on behalf of the COG-UK Consortium

**Affiliations:** 1 National Infection Service, Public Health England, London, United Kingdom; 2 MRC Centre for Global Infectious Disease Analysis, Jameel Institute for Disease and Emergency Analytics, Imperial College London, St Mary’s Campus, London, United Kingdom; 3 https://www.cogconsortium.uk

**Keywords:** Epidemiology, COVID-19, Infectious disease

## Abstract

**Background:**

The emergence of the SARS-CoV-2 Alpha variant in England coincided with a rapid increase in the number of PCR-confirmed COVID-19 cases in areas where the variant was concentrated.

**Aim:**

Our aim was to assess whether infection with Alpha was associated with more severe clinical outcomes than the wild type.

**Methods:**

Laboratory-confirmed infections with genomically sequenced SARS-CoV-2 Alpha and wild type between October and December 2020 were linked to routine healthcare and surveillance datasets. We conducted two statistical analyses to compare the risk of hospital admission and death within 28 days of testing between Alpha and wild-type infections: a matched cohort study and an adjusted Cox proportional hazards model. We assessed differences in disease severity by comparing hospital admission and mortality, including length of hospitalisation and time to death.

**Results:**

Of 63,609 COVID-19 cases sequenced in England between October and December 2020, 6,038 had the Alpha variant. In the matched cohort analysis, we matched 2,821 cases with Alpha to 2,821 to cases with wild type. In the time-to-event analysis, we observed a 34% increased risk in hospitalisation associated with Alpha compared with wild type, but no significant difference in the risk of mortality.

**Conclusion:**

We found evidence of increased risk of hospitalisation after adjusting for key confounders, suggesting increased infection severity associated with the Alpha variant. Rapid assessments of the relative morbidity in terms of clinical outcomes and mortality associated with emerging SARS-CoV-2 variants compared with dominant variants are required to assess overall impact of SARS-CoV-2 mutations.

## Background

Following the first wave of severe acute respiratory syndrome coronavirus 2 (SARS-CoV-2) infections in England, a second wave occurred in October and November 2020, with a transient decline in late November following a national lockdown. However, transmission accelerated in December 2020, coinciding with the emergence of a new SARS-CoV-2 variant initially concentrated in the South East, East of England and London regions.

At the time of the analysis, in February 2021, in the midst of the second wave, the total number of confirmed coronavirus disease (COVID-19) cases in England from the start of the pandemic had exceeded 4 million with over 114,000 people dying within 28 days of a positive test, the measure used in the United Kingdom (UK) to reflect current trends in deaths with SARS-CoV-2 infection.

This new variant was defined by 23 mutations: 13 non-synonymous mutations (including spike protein), four deletions and six synonymous mutations (five in ORF1ab: C913T, C5986T, C14676T, C15279T, C16176T, and one in the M gene: T26801C) [1]. Accordingly, this variant of concern (VOC) was initially assigned VOC 202012/01 as per standardised nomenclature [1]. At the time of emergence, before the establishment of the World Health Organization (WHO) variant naming system in May 2021 [2,3], this new variant was also known as the Kent variant, 20I/501Y.V1, or Phylogenetic Assignment of Named Global Outbreak LINeages (Pangolin) designation B.1.1.7 but was later designated as the Alpha variant by the WHO.

Consideration of this variant’s mutations including the spike protein mutation raised the possibility of changes to transmissibility. A national risk assessment considered evidence which indicated that this variant had greater transmissibility compared with wild-type variants in England [1,4].

Mathematical modelling at time of emergence indicated that the Alpha variant was associated with an increased reproduction number [5]. However, a broader public health risk assessment including information on health outcomes was required. To gain insights into the pathogenicity of the new Alpha variant compared with the globally circulating wild type, we assessed the outcomes associated with this variant in terms of hospital admission and mortality among people infected with genomically confirmed Alpha variant.

The aim of these analyses was to compare the risk of hospital admission within 14 days, and risk of death within 28 days of a positive SARS-CoV-2 test in cases infected with Alpha and wild-type SARS-CoV-2.

## Methods

### Selection of genomically sequenced SARS-CoV-2 isolates and data linkage

The UK Health Security Agency (UKHSA; formerly Public Health England) collates COVID-19 case data as part of the routine monitoring of COVID-19 cases in England.

Both positive PCR tests, reported by testing laboratories, and lateral flow device (LFD) tests, self-reported by testing individuals, are collected in the Second-Generation Surveillance System (SGSS), the national microbiology data repository at the UKHSA which includes statutory notifiable diseases such as COVID-19. Cases in this analysis were at person level and specimen dates relate to an individual’s first positive SARS-CoV-2 test. People with wild-type and Alpha SARS-CoV-2 variant infections were identified from whole genome sequencing (WGS) of PCR-confirmed tests which is nationally coordinated by the COG-UK consortium and uploaded to the Cloud Infrastructure for Big Data Microbial Bioinformatics (CLIMB) database [6]. At the point of analysis, widespread community PCR testing was available in England and ca 6% of laboratory-confirmed cases in England were genomically sequenced; however, positive SARS-CoV-2 specimens were selected for sequencing through a combination of targeted sequencing of key populations (recent travellers, hospitalised patients, contacts of genomically confirmed cases through enhanced case finding) and random selection. Confirmed cases with the Alpha variant were defined as sequenced PCR-positive cases who met the sequence definition for a confirmed Alpha variant [3] with a specimen date between 1 October and 31 December 2020. We classified as confirmed wild-type infections all infections with a sequence distinct from the Alpha variant or other known variants at that time.

Genomic data for Alpha and wild-type infections were linked to case data from SGSS. Demographic and clinical information, such as age, sex, ethnicity, residential address and symptom status at the time of test request was extracted. Address matching against national reference databases (Ordnance Survey AddressBase Premium) was undertaken to validate addresses, assign a Unique Property Reference Number and identify property type category: residential dwelling, care/nursing home and other property classifications (including prisons, residential institutions and homeless).

To assess hospitalisation among the cases with Alpha and wild-type infection, we linked the sequenced isolates (i) to National Health Service (NHS) hospital admission data from the NHS Digital Secondary Uses Service (SUS) [7], a repository of timely patient-level data for planning and commissioning healthcare services and clinical audit purposes, and (ii) to data on hospital admissions following emergency care attendance from the Emergency Care Data Set, the national dataset for urgent and emergency care. We analysed SUS data based on data available at 31 January 2021, to account for reporting delays in hospital admission data in SUS. To assess severity of disease, hospitalisation was defined as an admission to hospital within 14 days following a positive SARS-CoV-2 test.

We grouped admissions data into periods of continuous inpatient spells (CIP) based on the start and end dates of care episodes. Cases were matched to the nearest CIP within 14 days after the sample specimen date, those tested on the same day as or after admission were not considered a hospital admission. The end of a hospital stay was considered the date of discharge or date of death during hospitalisation.

To assess mortality, both Alpha variant and wild-type infections were linked to the UKHSA COVID-19 mortality dataset [8] to determine if cases had died as per data available at 17:00 on 8 February 2021, to account for 28 days to have elapsed since the included individual’s first positive specimen. These data record deaths in persons within 28 days following a laboratory-confirmed SARS-CoV-2 infection in England and are identified by matching confirmed COVID-19 cases to death reports from four sources: (i) hospital deaths reported by NHS England (ii) deaths recorded on the NHS Spine (national electronic health record database) identified through demographic batch service tracing (iii) death registrations from the Office for National Statistics and (iv) deaths reported by local Health Protection Teams related to local public health enquiries.

### Statistical analysis

We conducted two statistical analyses to compare the risk of hospital admission within 14 days and the risk of death within 28 days of a positive SARS-CoV-2 test between cases infected with Alpha and wild-type: (i) a matched cohort study matching cases with the Alpha variant to cases with the wild type and (ii) an adjusted Cox proportional hazards model to assess the association among all Alpha and wild-type infections during the study period, without limiting to those meeting the matching criteria of the matched cohort study.

In the matched cohort study, cases with the Alpha variant were frequency matched to cases with wild-type SARS-CoV-2 using a one-to-one ratio on: age group (10-year age bands), sex, specimen date (grouped into 2-week periods) and geographical region of residence (upper tier local authority, a geographical division in England reflecting county council areas, responsible for different local services). These factors were selected to control for confounding and to account for known associations between age and sex and poor clinical outcomes and to ensure the sample size was adequate for analysis. Matching on time and geographical region minimised potential for confounding related to regional variation in the rapid rise in case rates and resulting system pressures in the NHS.

In the matched cohort study, we used chi-squared tests for trend to assess differences between Alpha and wild-type infections in terms of sex, age, ethnicity, NHS region of residence and residential property classification. We calculated the number and proportion of cases with Alpha and wild-type infection hospitalised within 14 days of a positive test and dead within 28 days. The median length of hospital stay and time to death was assessed and a Kruskal-Wallis test was conducted to assess differences. We used a forward stepwise method to construct the logistic regression models, including variables with a p value of < 0.05 from the univariable analysis and a priori confounders; likelihood ratio tests were used to assess variables for which addition led to significant changes in the odds ratio (OR) for the adjusted model. The final adjusted logistic regression models were used to estimate the difference in the odds of hospitalisation and death among Alpha compared with wild-type infections.

Because the number of potential wild-type control cases was continually limiting as the Alpha variant became endemic in England, the number of cases included in the matched cohort analysis were limited and thus some cases with Alpha were excluded from this analysis. Therefore, we conducted a time-to-event analysis using the entire sequenced dataset to assess outcomes in terms of hospitalisation and death. Time-to-event analyses consisting of two Cox proportional hazards models were performed: (i) risk of hospitalisation within 14 days of a positive test and (ii) death within 28 days of a positive test. In each analysis, a single variable model was run for potential confounders: sex, age, ethnicity, NHS region of residence, residential property classification and week of specimen date. These confounders were chosen to reflect variations between the emerging Alpha variant and wild-type infections in terms of demographic factors and geographical distribution, and specimen date was included to account for the rapid rise in cases with the Alpha variant during the study period. Testing pillar was also included as a potential confounder: Pillar 1 included testing in UKHSA laboratories and NHS hospitals for those with a clinical need and for health and care workers, broadly capturing those with more severe illness, whereas Pillar 2 included swab testing available to the wider population, such as community testing sites and postal tests.

Multivariable proportional Cox regression models were also built using the forward stepwise approach and each potential confounder was considered using likelihood ratio tests. Age, sex, ethnicity and time (week of specimen) were included in the model as a priori variables.

## Results

### Matched cohort analysis

Among 63,609 sequenced confirmed COVID-19 cases, we identified 6,038 confirmed Alpha variant infections. The cohort analysis included 2,821 cases with Alpha matched to 2,821 cases with SARS-CoV-2 wild type. We excluded 3,217 cases with Alpha from the matched cohort analysis because of a lack of matching wild-type infections. These excluded cases differed from those included in the analysis in terms of age, geography and symptomatic status: excluded cases were more likely to be under 20 and over 60 years-old (chi-squared test overall: 133.9; p = 0.00), resident in London (chi-squared: 115.4; p = 0.00) and reporting symptoms (chi-squared: 26.9; p = 0.00).

In the matched cohort study, the median age of cases with the Alpha variant was 37 years and 52.9% were female (Table 1); they were predominantly resident in London (35.2%), the South East (29.2%) and East of England (20.4%). Comparator matching successfully selected a similar profile of wild-type infections (Table 1).

**Table 1:** Demographic characteristics of cases with SARS-CoV-2 Alpha or wild-type infection included in the matched cohort study, England, 1 October –31 December 2020 (n = 5,642)

	Alpha		Wild type		chi-square	p value
	n	%	n	%		
Total	2,821		2,821			
**Sex**						
Female	1,492	52.9	1,492	52.9	0.00	1.00
Male	1,329	47.1	1,329	47.1		
**Age (years)**						
< 10	98	3.5	98	3.5	0.00	1.00
10–19	362	12.8	362	12.8		
20–29	551	19.5	551	19.5		
30–39	571	20.2	571	20.2		
40–49	536	19.0	536	19.0		
50–59	440	15.6	440	15.6		
60–69	155	5.5	155	5.5		
70–79	47	1.7	47	1.7		
≥ 80	61	2.2	61	2.2		
**Ethnicity**						
Asian	330	11.7	410	14.5	13.9	0.02
Black	148	5.2	125	4.4		
Mixed	58	2.1	57	2.0		
Other	128	4.5	144	5.1		
Unknown	60	2.1	70	2.5		
White	2,097	74.3	2,015	71.4		
**NHSE Region**						
East of England	575	20.4	574	20.3	0.00	1.00
London	993	35.2	993	35.2		
Midlands	175	6.2	174	6.2		
North East and Yorkshire	111	3.9	112	4.0		
North West	105	3.7	106	3.8		
South East	825	29.2	826	29.3		
South West	37	1.3	36	1.3		
**Residential category**						
Care/nursing home	29	1	28	1	2.9	0.89
House in multiple occupancy	17	0.6	13	0.5		
Medical facilities (including hospitals, hospices and mental health clinics)	2	0.1	3	0.1		
Other property classifications	21	0.7	25	0.9		
Prisons, detention centres, secure units	8	0.3	13	0.5		
Residential dwelling (including houses, flats, sheltered accommodation)	2,679	95.0	2,672	94.7		
Residential institution (including residential education)	6	0.2	9	0.3		
Undetermined	59	2.1	58	2.1		
**Presence of symptoms**						
No	325	11.5	294	10.4	8.3	0.02
Yes	2,252	79.8	2,223	78.8		
Unknown	244	8.6	304	10.8		

NSHE: National Health Service England; SARS-CoV-2: severe acute respiratory syndrome coronavirus 2.

Cases infected with the Alpha variant were predominately of White ethnicity (74.3%) followed by Asian (11.7%) and Black (5.2%) ethnic groups (based on the classification in [9]). The ethnic profile of cases with wild-type infection was broadly similar but included fewer people of White ethnicity and more of Asian ethnicity (chi-squared: 13.9; p = 0.02).

The majority of COVID-19 cases were resident at private residential dwellings (95.0% for Alpha and 94.7% for wild-type) and there was no difference between Alpha and wildtype infections with regards to residential classification (chi-squared: 2.9; p = 0.89).

Four in five cases with Alpha (79.8%) and wild-type infection (78.8%) self-reported symptoms at the time of test (chi-squared: 8.3; p = 0.02).

### Assessment of hospitalisation

Of the 5,642 cases included in the matched cohort study, 131 individuals had a record of hospital admission within 14 days of the date of specimen collection: 76 (2.7%) in the Alpha and 55 (1.9%) in the wild-type group (chi-squared: 3.46; p = 0.006). The median age of the hospitalised cases with Alpha was 56 years (interquartile range (IQR): 47–65.5; range: 20–97) compared with 55 years for the wild type (IQR: 45–66; range: 0–85). A higher proportion of hospitalised people were male for both Alpha (43/76) and wild-type (31/55 infections (Table 2).

**Table 2:** Demographic characteristics of cases with SARS-CoV-2 Alpha or wild-type infection included in the matched cohort study who were hospitalised within 14 days of specimen date, England, 1 October –31 December 2020 (n = 131)

	Alpha		Wild type		chi-square	p value
	n	%	n	%		
**Hospitalised**						
Yes	76	2.7	55	1.9	3.45	0.06
No	2,745	97.3	2,766	98.1		
**Total**	**2,821 **		**2,821 **			
**Sex**						
Female	34	44.7	24	43.6	0.02	0.90
Male	42	55.3	31	56.4		
**Age (years)**						
< 10	0	0.0	2	3.6	8.67	0.28
10–19	0	0.0	0	0.0		
20–29	4	5.3	0	0.0		
30–39	7	9.2	5	9.1		
40–49	12	15.8	11	20.0		
50–59	28	36.8	16	29.1		
60–69	9	11.8	10	18.2		
70–79	4	5.3	5	9.1		
≥ 80	12	15.8	6	10.9		
**Ethnicity**						
Asian	6	7.9	5	9.1	1.96	0.74
Black	3	3.9	2	3.6		
Mixed	0	0.0	1	1.8		
Other	5	6.6	2	3.6		
Unknown	0	0.0	0	0.0		
White	62	81.6	45	81.8		
**NHSE Region**						
East of England	19	25.0	15	27.3	6.57	0.36
London	20	26.3	22	40.0		
Midlands	3	3.9	1	1.8		
North East and Yorkshire	4	5.3	5	9.1		
North West	4	5.3	2	3.6		
South East	25	32.9	10	18.2		
**Residential category**						
Care/nursing home	8	10.5	5	9.1	0.07	0.79
House in multiple occupancy	0	0.0	0	0.0		
Medical facilities (including hospitals, hospices and mental health clinics)	0	0.0	0	0.0		
Other property classifications	0	0.0	0	0.0		
Prisons, detention centres, secure units	0	0.0	0	0.0		
Residential dwelling (including houses, flats, sheltered accommodation)	68	89.5	50	90.9		
Residential institution (including residential education)	0	0.0	0	0.0		
Undetermined	0	0.0	0	0.0		
**Presence of symptoms**						
No	8	10.5	5	9.1	2.49	0.29
Yes	57	75.0	36	65.5		
Unknown	11	14.5	14	25.5		

NSHE: National Health Service England; SARS-CoV-2: severe acute respiratory syndrome coronavirus 2.

There was no significant association between Alpha variant and hospitalisation within 14 days including after adjusting for potential confounders (OR: 1.39; 95% confidence interval (CI) 0.98–1.98; p = 0.07). The length of hospital stay was similar between patients in the Alpha and wild-type groups (median length of stay: 5 days (IQR: 3–10; range: 0–37) vs 8 days (IQR: 4–13.5; range: 0–31), respectively; Kruskal Wallis p = 0.07).

### Time-to-event analysis

In the time-to-event analysis of 63,609 sequenced confirmed COVID-19 cases, 60,510 (95.1%) cases had hospitalisation data and complete demographic information and were therefore included in analysis. A total of 1,147 cases were hospitalised, representing 1.90% of all these cases, 120 (2.02%) with Alpha and 1,027 (1.88%) with wild-type infection (Table 3). The 60,510 included cases contributed a total of 838,211 days of follow-up time. In univariable analysis, there was no evidence of a statistical association between infection with the Alpha variant and risk of hospitalisation within 14 days (hazard ratio (HR): 1.07; 95%CI: 0.89–1.29; p = 0.48). However, after adjusting for potential confounders (sex, age, ethnicity, residential property classification and week of specimen date), we found a statistically significant association between hospitalisation and being infected with the Alpha variant, with the risk of hospitalisation 1.24 times higher among those for the Alpha variant compared with the wild type (HR: 1.34; 95% CI: 1.07–1.66; p = 0.01). Overall, hospitalisation was significantly associated with male sex, older age, non-white ethnicity, living in a care or nursing home or other non-residential property type, and with the week of specimen date (Table 3). In stratified analysis, there was no differential effect of the testing pillar on risk of hospitalisation (data not shown).

**Table 3:** Cox proportional hazards model to test hospitalisation within 14 days specimen date among all sequenced COVID-19 cases, England, 1 October –31 December 2020 (n = 60,510)

	All cases		Hospitalised cases		Univariate			Multivariate		
	n	% of total	n	% of cases	Hazard ratio	95% CI	p value	Hazard ratio	95% CI	p value
Alpha										
No	54,557	90.2	1,027	1.9	1.0	Reference		1.0	Reference	
Yes	5,953	9.8	120	2.0	1.1	0.9–1.3	0.48	1.3	1.1–1.7	0.01
Sex										
Female	31,850	52.6	507	1.6	1.0	Reference		1.0	Reference	
Male	28,660	47.4	640	2.2	1.4	1.3–1.6	0.00	1.4	1.2–1.6	0.00
Age (years)										
< 40	32,706	54.1	199	0.6	0.4	0.3–0.5	0.00	0.4	0.4–0.5	0.00
40–49	9,239	15.3	133	1.4	1.0	Reference		1.0	Reference	
50–59	8,875	14.7	254	2.9	2.0	1.6–2.5	0.00	2.1	1.7–2.6	0.00
60–69	4,658	7.7	204	4.4	3.1	2.5–3.9	0.00	3.2	2.6–4.0	0.00
70–79	2,520	4.2	200	7.9	5.7	4.6–7.1	0.00	6.0	4.8–7.5	0.00
≥ 80	2,512	4.2	157	6.3	4.5	3.5–5.6	0.00	4.6	3.6–5.9	0.00
Ethnicity										
Asian	8,104	13.4	180	2.2	1.2	1.0–1.4	0.03	1.8	1.5–2.1	0.00
Black	1,950	3.2	43	2.2	1.2	0.9–1.6	0.29	1.7	1.2–2.3	0.00
Mixed	1,068	1.8	16	1.5	0.8	0.5–1.3	0.38	1.6	1.0–2.7	0.05
Other	2,323	3.8	51	2.2	1.2	0.9–1.6	0.26	1.7	1.3–2.3	0.00
Unknown	1,502	2.5	5	0.3	0.2	0.1–0.4	0.00	0.3	0.1–0.7	0.00
White	45,563	75.3	852	1.9	1.0	Reference		1.0	Reference	
NHS region										
East of England	6,307	10.4	108	1.7	1.0	0.8–1.2	0.79	ND		
London	11,184	18.5	209	1.9	1.1	0.9–1.3	0.60			
Midlands	9,264	15.3	164	1.8	1.0	Reference				
North East and Yorkshire	13,144	21.7	280	2.1	1.2	1.0–1.5	0.06			
North West	12,123	20.0	225	1.9	1.1	0.8–1.3	0.65			
South East	6,498	10.7	118	1.8	1.0	0.8–1.3	0.83			
South West	1,990	3.3	43	2.2	1.2	0.9–1.7	0.24			
Residential property classification										
Care/nursing home	1,010	1.7	69	6.8	3.6	2.8–4.6	0.00	1.3	1.0–1.8	0.03
Residential	54,380	89.9	1,055	1.9	1.0	Reference		1.0	Reference	
Other property	5,120	8.5	23	0.5	0.2	0.2–0.4	0.00	0.3	0.2–0.5	0.00
Week										
40	1,938	3.2	21	1.1	1.0	Reference		1.0	Reference	
41	4,873	8.1	77	1.6	1.5	0.9–2.4	0.12	1.3	0.8–2.2	0.23
42	5,566	9.2	107	1.9	1.8	1.1–2.8	0.02	1.5	1.0–2.4	0.08
43	6,998	11.6	135	1.9	1.8	1.1–2.8	0.01	1.5	0.9–2.3	0.10
44	4,207	7.0	100	2.4	2.2	1.4–3.5	0.00	1.5	0.9–2.4	0.09
45	8,294	13.7	158	1.9	1.8	1.1–2.8	0.02	1.3	0.8–2.0	0.35
46	9,891	16.4	166	1.7	1.6	1.0–2.4	0.06	1.1	0.7–1.7	0.70
47	5,056	8.4	97	1.9	1.8	1.1–2.9	0.02	1.2	0.7–1.9	0.50
48	2,902	4.8	60	2.1	1.9	1.2–3.2	0.01	1.2	0.7–2.0	0.47
49	1,740	2.9	58	3.3	3.1	1.9–5.1	0.00	1.8	1.1–3.0	0.02
50	4,378	7.2	86	2.0	1.8	1.1–2.9	0.01	1.3	0.8–2.0	0.37
51	3,754	6.2	62	1.7	1.5	0.9–2.5	0.09	1.0	0.6–1.7	0.96
52	903	1.5	18	2.0	1.9	1.0–3.5	0.06	1.1	0.6–2.1	0.77
53	10	0.0	2	20.0	20.6	4.8–87.9	0.00	7.2	1.7–30.8	0.01

CI: confidence interval; COVID-19: coronavirus disease; ND: not done; NSHE: National Health Service England.

### Assessment of case fatality

Of the 5,642 cases included in the matched cohort study, a total of 76 individuals died within 28 days of a positive test; 36 (1.3%) with Alpha and 40 (1.4%) with wild type. (Table 4). The median age of cases who died with Alpha infection was 86 years (IQR: 82–91; range: 49–99) compared with 79 years for the wild type (IQR: 68–85; range: 48–97). Among those infected with the Alpha variant, 41.7% of deaths were in women compared with 32.5% of wild-type infections. In the matched cohort analysis, there was no evidence of an association between the Alpha or wild-type virus and death within 28 days of a positive SARS-CoV-2 specimen (OR: 0.90; 95% CI: 0.57–1.41; p = 0.64).

**Table 4:** Demographic characteristics of cases with SARS-CoV-2 Alpha or wild-type infection included in the matched cohort study who died within 28 days of specimen date, England, 1 October –31 December 2020 (n =76)

	Alpha		Wild type (comparator)		chi-square	p value
	n	%	n	%		
**Died**						
Yes	36	1.3	40	1.4	0.21	0.64
No	2,785	98.7	2,781	98.6		
**Total**	**2,821**		**2,821**			
**Sex**						
Female	15	41.7	13	32.5	0.68	0.41
Male	21	58.3	27	67.5		
**Age (years)**						
30–39	0	0.0	1	2.5	10.68	0.06
40–49	1	0.0	1	2.5		
50–59	1	2.8	4	10.0		
60–69	3	2.8	10	25.0		
70–79	3	8.3	7	17.5		
≥ 80	28	77.8	17	42.5		
**Ethnicity**						
Asian	2	5.6	3	7.5	0.12	0.94
Black	0	0.0	0	0.0		
Mixed	0	0.0	0	0.0		
Other	1	2.8	1	2.5		
Unknown	0	0.0	0	0.0		
White	33	91.7	36	90.0		
**NHSE Region**						
East of England	14	38.9	18	45.0	4.71	0.32
London	7	19.4	6	15.0		
Midlands	3	8.3	0	0.0		
North East and Yorkshire	2	5.6	1	2.5		
North West	0	0.0	0	0.0		
South East	10	27.8	15	37.5		
South West	0	0.0	0	0.0		
**Residential category**						
Care/nursing home	7	19.4	5	12.5	3.28	0.51
House in multiple occupancy	0	0.0	1	2.5		
Medical facilities (including hospitals, hospices and mental health clinics)	0	0.0	1	2.5		
Other property classifications	0	0.0	0	0.0		
Prisons, detention centres, secure units	0	0.0	0	0.0		
Residential dwelling (including houses, flats, sheltered accommodation)	29	80.6	32	80.0		
Residential institution (including residential education)	0	0.0	0	0.0		
Undetermined	0	0.0	1	2.5		
**Presence of symptoms**						
No	5	13.9	1	2.5	3.38	0.19
Yes	4	11.1	5	12.5		
Unknown	27	75.0	34	85.0		

NSHE: National Health Service England; SARS-CoV-2: severe acute respiratory syndrome coronavirus 2.

Among Alpha infections, the median time between positive specimen date and date of death was 8 days (IQR: 4–15; range: 2–26) compared with 9 days (IQR: 5–12; range: 2–26) among wild-type infections. There was no difference in distribution of time to death between cases infected with Alpha and wild-type (Kruskal Wallis p = 0.79).

In the time-to-event analysis of 63,609 genomically sequenced cases, 1,262 died within 28 days of their specimen date, 69 (1.2%) with Alpha and 1,189 (2.2%) with wild type (Table 5). Of the 63,609 sequenced cases, 61,051 were eligible for the time-to-event analysis and contributed 1,677,228 total days of follow up. Before adjusting for potential confounders, we observed a negative association between risk of death and infection with the Alpha variant (HR: 0.54; 95% CI: 0.42–0.69; p = 0.00). However, after adjusting for sex, age, ethnicity, residential property classification, week of specimen date and testing Pillar, this association was no longer observed, and there was no difference in risk of death when comparing Alpha with wild type (HR: 1.06; 95% CI: 0.82–1.38; p = 0.65). Overall, death was significantly associated with being male, older age, Asian ethnicity, living in a care or nursing home or other non-residential property type and with being tested in Pillar 1 (Table 5).

**Table 5:** Cox proportional hazards model to test death within 28 days specimen date among all sequenced COVID-19 cases, England, 1 October –31 December 2020 (n = 61,051)

	Cases		Cases who died		Univariate			Multivariate		
	n	% of total	n	% of cases	Hazard ratio	95% CI	p value	Hazard ratio	95% CI	p value
Alpha										
No	55,067	90.2	1,198	2.2	1.0	Reference		1.0	Reference	
Yes	5,984	9.8	69	1.2	0.5	0.4–0.7	0.00	1.1	0.8–1.4	0.65
Sex										
Female	32,096	52.6	551	1.7	1.0	Reference		1.0	Reference	
Male	28,955	47.4	716	2.5	1.5	1.3–1.6	0.00	1.7	1.5–1.9	0.00
Age (years)										
< 40	32,762	53.7	6	0.0	0.1	0.0–0.3	0.00	0.1	0.1–0.3	0.00
40–49	9,286	15.2	15	0.2	1.0	Reference		1.0	Reference	
50–59	8,971	14.7	62	0.7	4.09	2.3–7.2	0.00	4.0	2.3–7.1	0.00
60–69	4,767	7.8	141	3.0	18.1	10.6–0.8	0.00	14.0	8.2–23.9	0.00
70–79	2,610	4.3	316	12.1	80.8	48.1–135.6	0.00	39.3	23.2–66.3	0.00
≥ 80	2,655	4.4	727	27.4	212.2	127.3–353.9	0.00	70.8	420.–119.4	0.00
Ethnicity										
Asian	8,168	13.4	90	1.1	0.5	0.4–0.6	0.00	1.5	1.2–1.9	0.00
Black	1,968	3.2	17	0.9	0.3	0.2–0.6	0.00	0.8	0.5–1.2	0.26
Mixed	1,074	1.8	8	0.7	0.3	0.2–0.6	0.00	1.3	0.6–2.6	0.5
Other	2,343	3.8	31	1.3	0.6	0.4–0.8	0.00	1.2	0.8–1.7	0.34
Unknown	1,517	2.5	22	1.5	0.5	0.3–0.8	0.01	0.6	0.4–1.0	0.04
White	45,981	75.3	1,099	2.4	1.0	Reference		1.0	Reference	
NHS Region										
East of England	6,393	10.5	215	3.4	1.6	1.3–1.9	0.00			
London	11,312	18.5	226	2.0	1.0	0.8–1.2	0.57			
Midlands	9,304	15.2	204	2.2	1.0	Reference				
North East and Yorkshire	13,263	21.7	198	1.5	0.7	0.6–0.9	0.00			
North West	12,239	20.1	310	2.5	1.2	1.0–1.4	0.07			
South East	6,548	10.7	100	1.5	0.7	0.6–0.9	0.01			
South West	1,992	3.3	14	0.7	0.3	0.2–0.6	0.00			
Residential property classification										
Care/nursing home	1,086	1.8	249	22.9	15.1	13.2–17.4	0.00	1.4	1.3–1.7	0.00
Residential	54,828	89.8	1,003	1.8	1.0	Reference		1.0	Reference	
Other property	5,137	8.4	15	0.3	0.2	0.1–0.3	0.00	0.4	0.2–0.6	0.00
Week										
40	1,943	3.2	19	1.0	1.0	Reference		1.0	Reference	
41	4,893	8.0	67	1.4	1.4	0.8–2.3	0.21	0.9	0.5–1.5	0.64
42	5,595	9.2	87	1.6	1.6	1.0–2.6	0.07	1.1	0.7–1.8	0.75
43	7,042	11.5	107	1.5	1.5	0.9–2.4	0.11	0.9	0.5–1.4	0.56
44	4,230	6.9	121	2.9	2.9	1.8–4.7	0.00	1.1	0.7–1.7	0.78
45	8,344	13.7	140	1.7	1.7	1.1–2.8	0.03	0.9	0.5–1.4	0.51
46	9,978	16.3	187	1.9	1.9	1.2–3.0	0.01	0.9	0.5–1.4	0.49
47	5,128	8.4	123	2.4	2.4	1.5–3.9	0.00	0.9	0.5–1.4	0.52
48	2,979	4.9	110	3.7	3.7	2.3–6.1	0.00	0.9	0.5–1.4	0.51
49	1,779	2.9	80	4.5	4.7	2.8–7.7	0.00	1.0	0.6–1.7	0.99
50	4,411	7.2	93	2.1	2.1	1.3–3.5	0.00	0.9	0.5–1.5	0.67
51	3,796	6.2	74	2.0	2.0	1.2–3.3	0.01	0.9	0.5–1.5	0.69
52	922	1.5	57	6.2	6.5	3.9–10.9	0.00	1.4	0.8–2.4	0.19
53	11	0.0	2	18.2	20.3	4.7–86.9	0.00	1.5	0.4–6.6	0.57
Pillar										
1	7,078	11.6	1,001	14.14	31.4	27.44–36.0	0.00	5.6	4.8–6.5	0.00
2	5,3973	88.4	266	0.49	1.0	Reference		1.0	Reference	

CI: confidence interval; COVID-19: coronavirus disease; ND: not done; NSHE: National Health Service England.

## Discussion

Following its identification, the SARS-CoV-2 Alpha variant became a focus of international interest, with cases identified worldwide following its initial detection in England [10]. During the study period (October to December 2020), England had the highest number of genomically confirmed cases of this variant internationally [11]. Vaccination against SARS-CoV-2 started at the end of 2020, so this analysis mainly reflects a period when the majority of the population was not protected by vaccination.

Early risk assessments of the emerging Alpha variant by public health agencies and independent scientific groups suggested increased transmissibility based on mathematical modelling and the rapid increases in cases where the variant was first concentrated [12,13]. Early reports, from January 2021, further suggested that the variant may be associated with an increased risk of mortality [14].

This early study demonstrated an increased hospitalisation risk (HR: 1.34; 95% CI: 1.07–1.66) for genomically confirmed COVID-19 cases with the Alpha variant following adjustment for key risk factors such as ethnicity and specimen collection date; it was the first to individually link hospitalisation records nationally to cases with the Alpha variant in England and provided an improved level of evidence for public health decision-making. Analysis of hospitalisation risk in England associated with the Alpha variant before this were either ecological assessments (for sequence-confirmed Alpha infections without individual-level record linkage) [14] or reliant on the use of a proxy measure of S gene target failure (SGTF) to identify the variant, whereby diagnostic PCR tests can detect the Alpha variant through failed PCR amplification of the spike gene target but preserved detection of other targets. Although we studied a period where a high proportion of SGTF cases were verified as Alpha, the use of varying SGTF case definitions globally mean that sequence confirmation remains the gold standard for these analyses.

An additional advantage of this analysis is that we included cases detected through both mass testing in the community (tests captured in Pillar 2) and individuals tested in hospital settings (tests captured in Pillar 1) who were more likely to be at risk of severe COVID-19 presentations such as elderly people and those with co-morbidities. All previous analyses in England identified cases infected with the Alpha variant through SGTF and were therefore restricted to cases captured exclusively in Pillar 2, where individuals are likely to be less unwell, subjecting these analyses to bias in terms of estimating severity.

Notably, the majority of the sequenced cases were initially identified through mass population testing and both Alpha and wild-type groups had similar proportions of asymptomatic infection at time of testing, suggesting initial presentations are similar for both groups.

We were unable to demonstrate an increased risk of death through either the Cox proportional hazard model or the matched cohort design whereas this has been observed via SGTF-based analysis elsewhere [15]. This may be explained by the difference in scale, with SGTF-based analyses being far larger in case numbers than analysis using sequence-confirmed cases, and therefore the former will have greater power to detect smaller differences.

These analyses presented here were made possible by the large-scale, rapid identification of the Alpha variant and wild-type infections via the national COG-UK collaboration which has conducted large-scale WGS of SARS-CoV-2 throughout the COVID-19 pandemic.

Because there was an urgent need for a risk assessment of the Alpha variant following its detection, these analyses were limited by short follow-up time and relatively small numbers of individuals experiencing the outcomes assessed. This was managed by standardising the follow-up time after positive specimen date to within 14 days for hospitalisations and within 28 days for case fatalities. In addition, we did not have data for this analysis relating to the date of symptom onset nor additional data on co-morbidities, therefore these could not be included in this analysis.

The use of routine admissions data designed for administrative purposes caused potential delays in identifying and reporting such signals, and these numbers are likely to represent a minimum estimate.

## Conclusion

Emerging SARS-CoV-2 variants add complexity to the pandemic response, with potential differences in disease severity and transmissibility. Rapid and systematic assessments of severe outcomes associated with emerging variants, as described here, have become an essential part of the pandemic response. These assessments, alongside assessment of the transmissibility of emerging variants and vaccine effectiveness against these variants, are essential to inform the control of COVID-19.
